# Blood calprotectin as a biomarker for infection and sepsis – the prospective CASCADE trial

**DOI:** 10.1186/s12879-024-09394-x

**Published:** 2024-05-16

**Authors:** Eva Diehl-Wiesenecker, Noa Galtung, Johannes Dickescheid, Monika Prpic, Rajan Somasundaram, Kai Kappert, Wolfgang Bauer

**Affiliations:** 1 Department of Emergency Medicine, Charité – Universitätsmedizin Berlin, corporate member of Freie Universität Berlin and Humboldt-Universität zu Berlin, Benjamin Franklin Campus, Zentrale Notaufnahme und Aufnahmestation, Hindenburgdamm 30, 12203 Berlin, Germany; 2 Institute of Diagnostic Laboratory Medicine, Clinical Chemistry and Pathobiochemistry, Charité – Universitätsmedizin Berlin, corporate member of Freie Universität Berlin and Humboldt-Universität zu Berlin, Augustenburger Platz 1, 13353 Berlin, Germany; 3 Labor Berlin – Charité Vivantes GmbH, Berlin, Germany

**Keywords:** S100A8, S100A9, Leukocyte L1 Antigen Complex, Acute, Bacterial, Emergency Department, Serum, Plasma, Diagnostic, Prognostic

## Abstract

**Background:**

Early in the host-response to infection, neutrophils release calprotectin, triggering several immune signalling cascades. In acute infection management, identifying infected patients and stratifying these by risk of deterioration into sepsis, are crucial tasks. Recruiting a heterogenous population of patients with suspected infections from the emergency department, early in the care-path, the CASCADE trial aimed to evaluate the accuracy of blood calprotectin for detecting bacterial infections, estimating disease severity, and predicting clinical deterioration.

**Methods:**

In a prospective, observational trial from February 2021 to August 2022, 395 patients (*n* = 194 clinically suspected infection; *n* = 201 controls) were enrolled. Blood samples were collected at enrolment. The accuracy of calprotectin to identify bacterial infections, and to predict and identify sepsis and mortality was analysed. These endpoints were determined by a panel of experts.

**Results:**

The Area Under the Receiver Operating Characteristic (AUROC) of calprotectin for detecting bacterial infections was 0.90. For sepsis within 72 h, calprotectin’s AUROC was 0.83. For 30-day mortality it was 0.78. In patients with diabetes, calprotectin had an AUROC of 0.94 for identifying bacterial infection.

**Conclusions:**

Calprotectin showed notable accuracy for all endpoints. Using calprotectin in the emergency department could improve diagnosis and management of severe infections, in combination with current biomarkers.

**Clinical trial registration number:**

DRKS00020521

**Supplementary Information:**

The online version contains supplementary material available at 10.1186/s12879-024-09394-x.

## Background

Acute infections and sepsis are among the leading causes of in-hospital deaths worldwide [1]. Among patients with sepsis, 90% of cases develop outside of hospital [2]. These patients are often first seen in the emergency department (ED), where key treatment decisions must be made. Even infections presenting with mild signs and symptoms may rapidly deteriorate [3, 4]. Making these treatment decisions quickly, especially regarding antibiotic therapy and level of care, can have profound implications on the outcomes of these patients, leading to the introduction of sepsis bundles to standardize care and encourage ED physicians to rapidly begin treatment [5]. However, this often comes at a cost of inaccurate treatment [6–8]. Biomarkers therefore play a crucial role in supporting ED physicians in early diagnostic and treatment decisions [6, 9, 10].

Calprotectin, a calcium-binding protein released by neutrophils, is a key early alarmin in the host immune response to infection and generates a pro-inflammatory feedback loop [11–13]. While already established as a biomarker in stool for chronic inflammatory bowel disease, blood-calprotectin showed promise in observational studies, mainly in ICU settings, as a marker for acute infections and sepsis [14–16], especially in the early stages of infection and deterioration [17]. Several studies have demonstrated the value of calprotectin in predicting multi-organ failure in severe infections and sepsis [14, 15, 18, 19]. Recent studies in animal models have also found potential therapeutic benefits of inhibiting calprotectin pharmacologically [20]. As sepsis is predicated on a hyperinflammatory host response, blood calprotectin may be a novel marker for early detection of sepsis, severe infections, and prediction of clinical deterioration.

Currently, the diagnostic and subsequent therapeutic management of patients with suspected infection and/or sepsis depends on the physician’s experience, which is supported by a limited number of clinically established and available biomarkers, notably C-reactive protein (CRP) and procalcitonin [21–24]. Although these parameters may play an important role as markers of inflammation, both lack the specificity to be used as a gold standard biomarker for sepsis. Novel biomarkers and multi-biomarker algorithms have shown promising results, yet integration into the clinical routine is slow, in part due lengthy assay development and regulatory approval processes [25]. Further, serum lactate concentrations are often used as an indicator of shock and therefore of infection severity. However, lactate is not sufficiently specific to be used as a single marker of severity and deterioration from infection to sepsis [26].

To evaluate if blood calprotectin could add value to the routinely used biomarkers, we conducted the CASCADE (“**C**alprotectin in **A**cute Infections and **S**epsis for Prognosis, **C**haracterisation, **a**nd **D**iagnosis in the **E**mergency Department”) trial, the first large-scale, prospective evaluation of calprotectin as a biomarker for bacterial infections and sepsis in the emergency department, combining infection identification and estimation of the disease outcome and severity.

## Methods

### Study design and patient selection

**Fig. 1:**
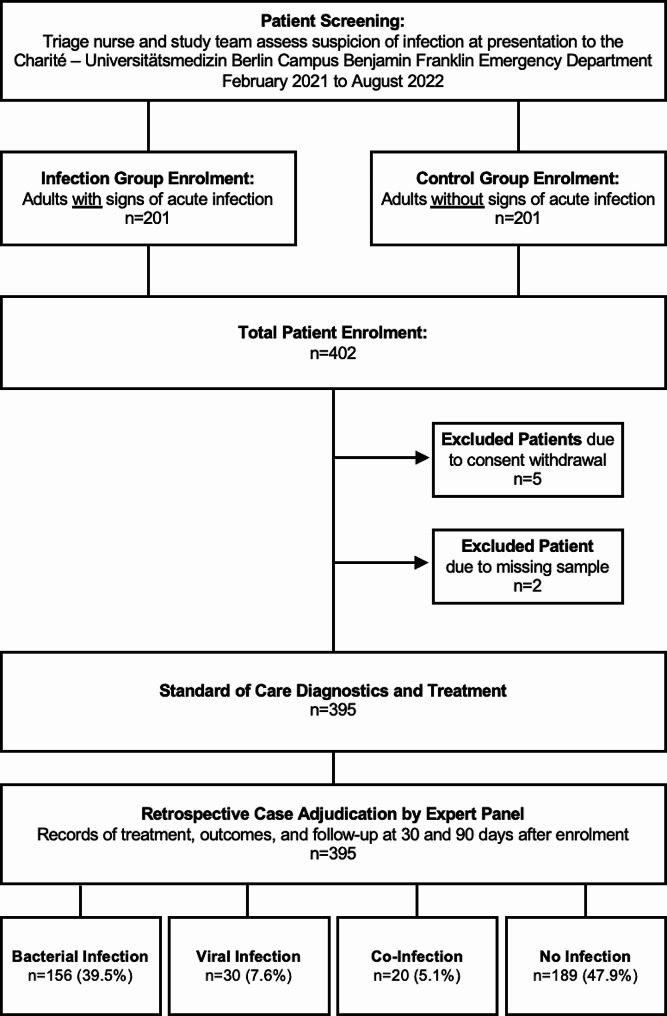
Screening, enrolment and adjudication flowchart. Patient enrolment flowchart for the CASCADE trial. In total 402 patients were enrolled, of which 395 remained after exclusions. Case adjudication by pairs of specialist physicians resulted in the final segmentation by infection status

We conducted a prospective, observational study at the emergency department of a tertiary care university hospital in Berlin, Germany from February 2021 to August 2022. The trial design is shown in Fig. 1. Briefly, adult patients (≥ 18 years) were screened and recruited by a trained team of nurses and physicians at the point of enrolment. Patients with a suspected acute infection were enrolled into the primary cohort, while age- and sex-matched patients presenting with a non-infectious condition were enrolled into the control cohort. The control cohort was enrolled at a similar rate to the primary cohort over the course of the study. Patients already diagnosed with an infection or sepsis, who had received systemic antibiotics prior to ED presentation, or who previously participated in this trial, were excluded. Patients were eligible irrespective of pre-existing conditions. Informed consent (of the patients and/or their proxies), vital signs, medical records, and blood samples were collected during enrolment. Patients unable to consent at this point were enrolled on an interim base until the consent was given retroactively or the patient had to be excluded. All patients were treated due to the standard procedure of the ED. The study was approved by the institutional review board (committee: https://ethikkommission.charite.de/en/, approval number: EA2-044-20) and registered with the German Clinical Trials Register (ID: DRKS00020521).

### Trial endpoints

The primary endpoint of the trial was the ability of calprotectin to differentiate bacterial infections from virally- and non-infected patients. Secondary endpoints included 30-day mortality and sepsis.

A clinical adjudication of each case was conducted once the patient was discharged from hospital and at least 30 days after enrolment, or after the patient died. Two senior physicians, trained in internal medicine and emergency medicine, retrospectively assessed each case, to independently evaluate the presence of a bacterial infection, a viral infection, and the occurrence of multi-organ dysfunction. For this process, the experts had access to the medical records, including vital signs, examination findings, radiological, microbiological and all laboratory data except calprotectin measurements, and the final report. Both experts made their assessment independently and compared the results afterwards. In the case of disagreement, the cases were discussed until consensus. For multi-organ dysfunction, physicians evaluated evidence of impaired function for each of the Sequential Organ Failure Assessment (SOFA) organ systems and noted the timepoint of impairment. Patients with two-or-more impaired organ systems at any time point within 72 h of presentation were considered to have multi-organ dysfunction. Patients who were adjudicated as both having an infection, as well as experiencing multi-organ dysfunction within 72 h were considered to have sepsis. While significant impairment of a single organ system is typically used to diagnose sepsis, the ED environment often is confronted by patient histories of pre-existing organ dysfunction being incomplete. As such, here we required dysfunction in two or more organ systems to avoid patients with unknown pre-existing dysfunctions being erroneously considered septic. Patients, their guardians, or primary care physicians were contacted 30 days after enrolment by telephone follow-up in order to assess mortality.

### Measurement of calprotectin

Blood collection Lithium Heparin vacuum tubes from Greiner Bio-One GmbH (Kremsmünster, Austria) were used. Plasma samples for calprotectin measurement were collected at the point of enrolment, centrifuged at 3,000 G for 10 min and measured on a cobas c501® within a cobas 8000® analyzer (both Roche, Basel, Switzerland) using the particle enhanced turbidimetric immunoassay (PETIA) (Gentian Diagnostics, Moss, Norway). In a subset (29 samples), measurements were done in serum samples from vacuum gel tubes due to a lack of available plasma samples. Using an additional sub-cohort of 30 patients with both serum and heparin plasma measurements, a linear regression analysis allowed for the calculation of a corrective equation to convert the serum concentrations of the 29 patients without plasma samples into plasma-equivalents for a better comparison of samples regardless of measured tube types. More details are shown in the supplemental methods.

### Statistical methods

The sample size was calculated on the basis of unpublished results from a small pilot study we performed in 2020 in the emergency department (*n* = 32, similar selection criteria and measurement methodology), combined with results from comparable other studies [27]. Using nQuery (Statsols, Boston, USA), the power analysis, based on a Wilcoxon-Mann-Whitney Test (effect size = 0.3, alpha = 5%, power = 80%), resulted in a total sample size of *n* = 368. Planning for a dropout rate of 5–8% resulted in a final sample size of approximately *n* = 400.

Continuous variables are presented with the median and interquartile range (IQR) and were compared using Mann-Whitney U test. Nominal variables are presented as n and column percentages and were compared using Fisher exact test. The accuracy of calprotectin to identify bacterial infections, and to identify and predict sepsis was analysed.

The performance of calprotectin without specified cut-off values is shown and compared with currently used biomarkers and scores for bacterial infection and severity (CRP, procalcitonin, lactate) by using the area under receiver operating characteristic (AUROC). AUROCs were compared using DeLong’s method. R-Studio Version 2023.06.1 (RStudio: Integrated Development for R. RStudio, PBC, Boston, MA) was used for the statistical analysis.

## Results

### Baseline characteristics and calprotectin measurements

We enrolled a consecutive cohort of *n* = 402 patients, consisting of 201 adult patients with clinically suspected infections and a further 201 adult patients without clinically suspected infections, used as a control group. All patients were enrolled at presentation to the emergency department between February 2021 and August 2022 (Fig. 1). Five patients were excluded due to missing or withdrawn consent, and a further two patients were excluded due to missing samples for calprotectin measurements, resulting in a final cohort of *n* = 395 patients. Patient characteristics are summarized by adjudicated infection status in Table 1. The calprotectin concentrations weakly correlated with those of procalcitonin (*R* = 0.17, *p* < 0.001) and CRP (*R* = 0.65, *p <* 0.001). The correlation plots are shown in supplemental Figure S1.

**Table 1:** Baseline Characteristics at enrolment and outcomes by adjudicated infection status. Continuous variables are shown as median and interquartile range, nominal variables are shown with frequency and column percentages. “Co-Infection” refers to patients with both a bacterial and viral infection. Abbreviations: BP = Blood pressure; CRP = C-reactive protein; SpO2 = Oxygen saturation; COPD = Chronic Obstructive Pulmonary Disease; MOD = Multi-Organ Dysfunction; ICU = Intensive Care Unit; WBC = White blood cell count

	Bacterial (*N* = 156)	Viral (*N* = 30)	Co-Infection (*N* = 20)	No Infection (*N* = 189)
**Enrolment Group**				
Suspected Infection	145 (92.9%)	21 (70.0%)	20 (100.0%)	8 (4.2%)
Control	11 (7.1%)	9 (30.0%)	0 (0.0%)	181 (95.8%)
**Demographics**				
Age [years]	77.0 (69.0, 83.0)	57.0 (38.0, 73.5)	75.0 (58.2, 85.0)	74.0 (64.0, 81.0)
Female	65 (41.7%)	11 (36.7%)	11 (55.0%)	86 (45.5%)
**Vital Signs**				
Respiratory Rate [/min]	20 (16, 26)	16 (15, 22)	20 (15, 24)	16 (14, 17)
Systolic BP [mmHg]	124 (105, 142)	132 (122, 142)	122 (104, 141)	145 (131, 160)
Temperature [°C]	37.8 (36.7, 38.9)	37.1 (36.5, 38.2)	37.0 (36.1, 38.1)	36.4 (36.1, 36.7)
Heart Rate [/min]	99 (85, 113)	84 (74, 102)	98 (83, 115)	78 (69, 92)
**Comorbidity**				
Malignancy	41 (26.3%)	2 (6.7%)	5 (25.0%)	42 (22.2%)
Type 2 Diabetes	39 (25.0%)	6 (20.0%)	4 (20.0%)	26 (13.8%)
COPD	18 (11.5%)	2 (6.7%)	2 (10.0%)	18 (9.5%)
Immunosuppression	17 (11.0%)	1 (3.3%)	4 (20.0%)	8 (4.2%)
**Biomarkers**				
Calprotectin [μg/mL]	6.9 (4.6, 10.3)	3.7 (2.4, 4.4)	4.5 (3.4, 6.6)	2.1 (1.5, 3.0)
Procalcitonin [μg/L]	0.8 (0.2, 3.2)	0.1 (0.1, 0.2)	0.4 (0.1, 1.2)	0.0 (0.0, 0.1)
CRP [mg/L]	125 (47.2, 205)	26.6 (2.6, 87.5)	62.1 (10.2, 177.0)	1.1 (0.7, 3.4)
WBC [/nL]	13.6 (9.8, 19.0)	7.1 (5.7, 8.9)	13.7 (9.7, 18.8)	7.6 (6.2, 8.9)
Lactate [mg/dL]	19.0 (13.0, 28.9)	14.0 (11.0, 17.5)	18.0 (11.8, 26.2)	13.0 (10.8, 17.0)
**Outcomes**				
MOD within 72 h	57 (36.5%)	1 (3.3%)	6 (30.0%)	1 (0.5%)
Sepsis within 72 h	57 (36.5%)	1 (3.3%)	6 (30.0%)	0 (0.0%)
ICU within 72 h	41 (26.3%)	5 (16.7%)	7 (35.0%)	10 (5.3%)
Death by day 30	28 (18.1%)	0 (0.0%)	0 (0.0%)	2 (1.1%)

The total median cohort age was 75 years (interquartile range (IQR) = 63–82 years) with 173 (43.8%) of participants being female. Our cohort included several patients with comorbidities (*n* = 90 (22.8%) with active malignant disease, *n* = 75 (19.0%) with type 2 diabetes, *n* = 30 (7.6%) immunosuppressed). For baseline characteristics by enrolment group (suspected infection vs. no suspected infection), see supplemental Table S1.

The expert adjudication of infection status classified 156 (39.5%) patients as having had a bacterial infection, 30 (7.6%) patients a viral infection, 20 (5.1%) patients a bacterial-viral co-infection, and 189 (47.8%) patients as having had no infection. Of these 189 patients with no infection, 181 (95.8%) were from the control group at enrolment, while 145 of 156 (93%) bacterial patients, 21 of 30 (70%) viral patients, and 20 of 20 (100%) co-infected patients were from the primary enrolment group.

### Diagnostic performance of calprotectin for bacterial infections

**Fig. 2:**
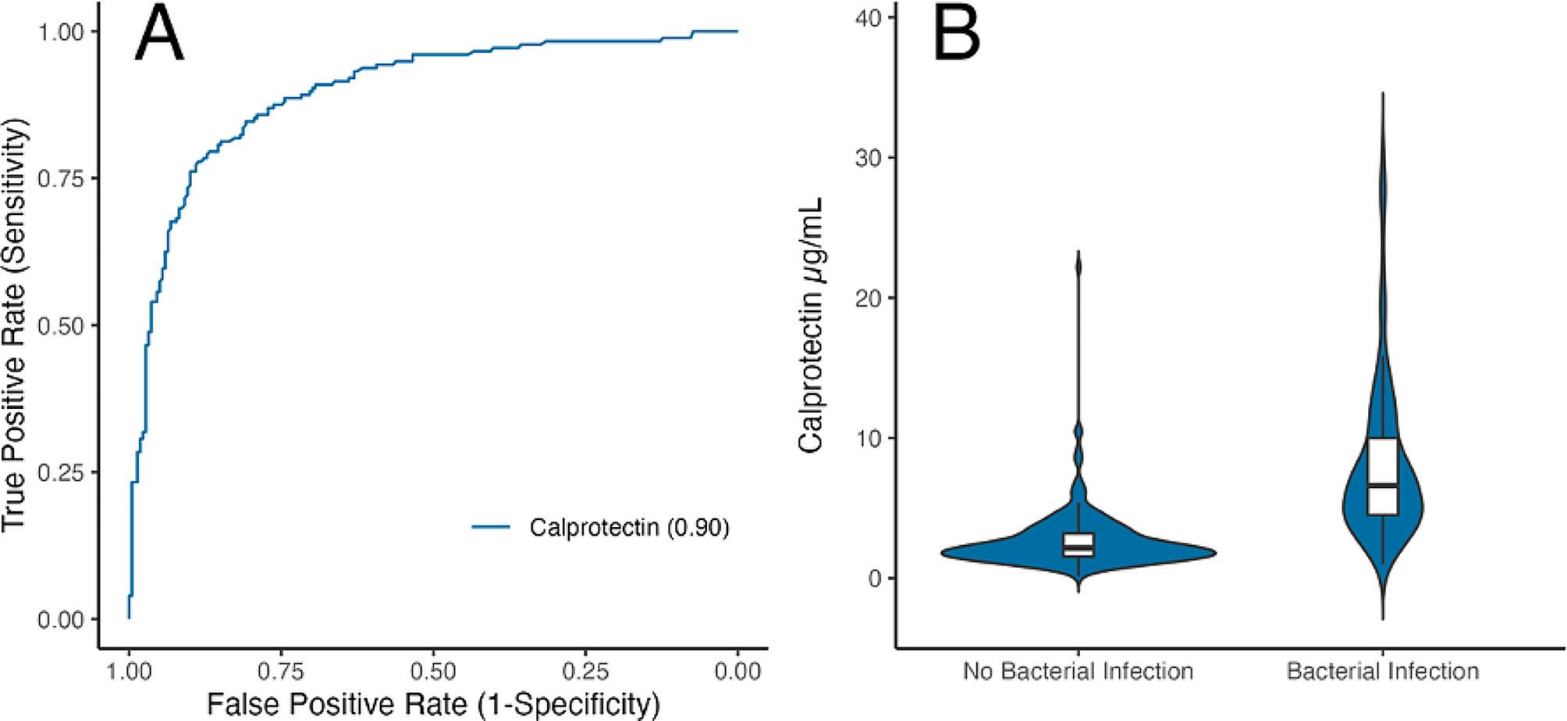
Calprotectin identifying bacterial infections. (**A**) ROC of calprotectin identifying bacterial infections and (**B**) Distribution of calprotectin among patients with and without bacterial infections. This includes bacterial-viral co-infections as bacterial, and both non-infectious and viral conditions as non-bacterial. Since C-reactive protein and procalcitonin were used in the adjudication of these endpoints the ROCs are not shown here as comparators. These ROCs are available in supplemental Figure S3

The median concentration of plasma calprotectin was 3.5 μg/mL (IQR = 2.0–6.4) across the cohort and varied significantly by adjudicated group, with the highest values in patients with solely bacterial infection, as shown in Table 2. The distributions of calprotectin by infection type are shown in supplemental Figure S2. For diagnosing bacterial infections (including co-infections), calprotectin had an AUROC of 0.90 (95% Confidence Interval (CI) = 0.86–0.93), as shown in Fig. 2. The AUROCs for procalcitonin and C-reactive Protein (CRP) were 0.94 (0.91–0.96) and 0.93 (0.91–0.96) respectively. Of note, both markers were used by the expert panel during the adjudication process and are thus not considered to be valid comparators for this endpoint.

**Table 2:** Table of calprotectin and procalcitonin concentrations in patients with key endpoints, shown with median and interquartile range. Additionally, a *p*-value, comparing the biomarker concentrations in the given group to the concentrations in patients without an infection (controls) using a Mann-Whitney U test is shown. * No *p*-value is shown comparing “No Sepsis” with “No Infection”, due to the overlap between these groups. Abbreviations: BSI = Bloodstream infection

Endpoint	Cases	Calprotectin [ug/mL]			Procalcitonin [ng/mL]		
		Median	IQR	*p*-value	Median	IQR	*p*-value
**Infection Status**							
Bacterial	156	6.89	4.61–10.29	< 0.001	0.76	0.20–3.22	< 0.001
Viral	30	3.67	2.39–4.40	< 0.001	0.08	0.05–0.16	< 0.001
Co-Infection	20	4.54	3.45–6.56	< 0.001	0.39	0.08–1.21	< 0.001
No Infection	189	2.10	1.51–3.01	-	0.04	0.03–0.06	-
**Bacterial Focus**							
Pulmonary	33	5.17	4.28–7.25	< 0.001	0.39	0.13–1.08	< 0.001
Urogenital	60	7.04	4.38–9.44	< 0.001	1.12	0.26–5.54	< 0.001
Abdominal	15	5.45	4.60–7.62	< 0.001	2.12	0.54–45.33	< 0.001
Skin & Soft Tissue	9	6.60	5.32–9.68	< 0.001	0.24	0.13–1.41	< 0.001
Orthopaedic	2	11.73	11.18–12.28	0.016	0.59	0.56–0.62	0.014
BSI Without Focus	4	9.02	4.99–12.44	0.004	2.41	2.00-51.70	0.003
Multiple Foci	46	7.08	4.39–10.51	< 0.001	0.78	0.19–2.11	< 0.001
Unclear Focus	3	10.40	7.67–10.66	0.004	0.19	0.18–0.86	0.004
Other Foci	4	9.32	6.78–15.35	0.001	0.66	0.47–6.84	0.002
**Sepsis**							
No Sepsis	332	3.00	1.81–4.85	*	0.07	0.04–0.24	< 0.001
Sepsis	63	8.44	5.83–12.25	< 0.001	1.33	0.58–14.70	< 0.001
**Blood Culture**							
Gram Positive	35	7.99	5.92–13.42	< 0.001	1.06	0.60–4.48	< 0.001
Gram Negative	55	7.18	5.02–11.28	< 0.001	2.46	0.70-24.12	< 0.001

### Bacterial infection sites

Of the 176 patients with a bacterial aetiology (including co-infections), the site of the bacterial infection is shown in Table 2. For all of the evaluated bacterial infection sites, calprotectin was significantly elevated compared to patients without infections. Among patients with positive blood cultures, when segmented by gram-staining results of bacteria identified, both gram-positive and gram-negative patients had similarly elevated calprotectin concentrations (7.99 μg/mL vs. 7.18 μg/mL, both *p* < 0.001 compared to no infection).

### Calprotectin in key subpopulations

In an exploratory analysis, the accuracy for calprotectin, CRP, and procalcitonin for diagnosing bacterial infections in key subpopulations, namely patients with immunosuppression, with diabetes, with renal failure, patients older than 75 years, and patients younger than 75 years, are shown in Table 3. In each of these subpopulations, the performance of calprotectin remained stable, with AUROCs ≥ 0.88. However, among patients with diabetes, the accuracy of calprotectin increased to an AUROC of 0.94 (95%-CI = 0.89–0.99), while that of procalcitonin and CRP decreased to 0.90 (0.83–0.97) and to 0.87 (0.78–0.95). The full plots are shown in supplemental Figure S4.

**Table 3:** Table of calprotectin, procalcitonin, and CRP diagnostic performance for identifying bacterial infections in key patient subpopulations. An individual patient can be included in more than one group shown in Table 3 and Figure S4. Performance is shown using the Area Under the Receiver Operating Characteristics (AUROCs) and 95% confidence intervals. For each subpopulation, the number of patients with and without bacterial infection is shown. Plots of the ROC curves are available in supplemental Figure S4. Abbreviations: CRP = C-reactive protein

Subpopulation	Bacterial Infection	Non-Bacterial	Calprotectin AUC (95% CI)	Procalcitonin AUC (95% CI)	CRP AUC (95% CI)
Immunosuppression	21	9	0.88 (0.76-1.00)	0.98 (0.94-1.00)	0.97 (0.92-1.00)
Diabetes	45	32	0.94 (0.89–0.99)	0.90 (0.83–0.97)	0.87 (0.78–0.95)
Renal Failure	53	8	0.88 (0.77–0.99)	0.96 (0.92-1.00)	1.00 (1.00–1.00)
Age ≥ 75 years	106	93	0.91 (0.87–0.95)	0.96 (0.93–0.98)	0.95 (0.92–0.98)
Age < 75 years	76	134	0.89 (0.84–0.94)	0.91 (0.87–0.96)	0.93 (0.89–0.96)

### Diagnostic performance of calprotectin for sepsis and mortality

**Fig. 3:**
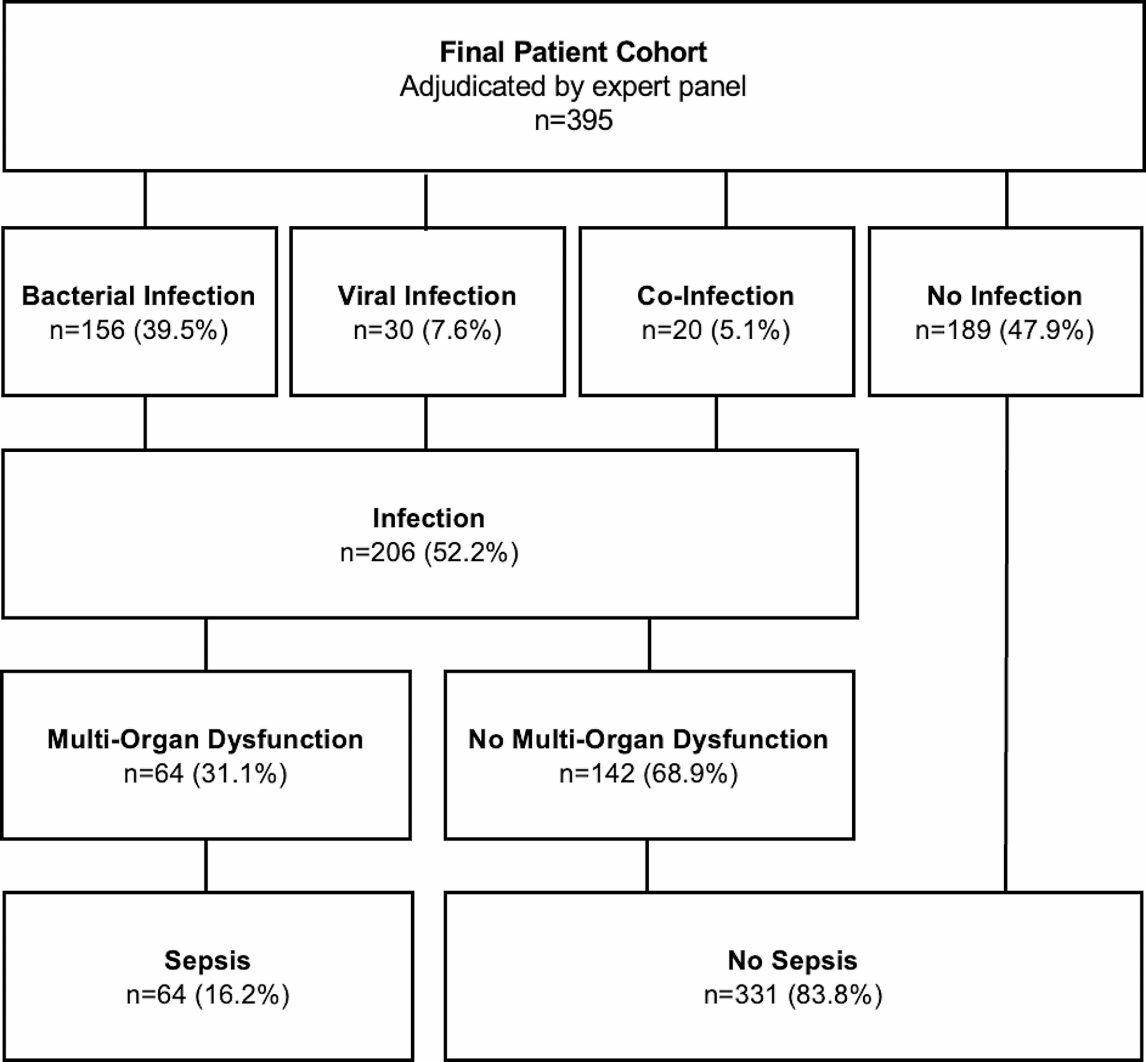
Multi-organ dysfunction and sepsis assessment. Patient adjudication flowchart explaining the trial definition of sepsis. Multi-organ dysfunction was assessed retrospectively via chart review using the Sequential Organ Failure Assessment organ systems. Patients adjudicated as having both an acute infection as well as multi-organ dysfunction were considered to have sepsis

**Fig. 4:**
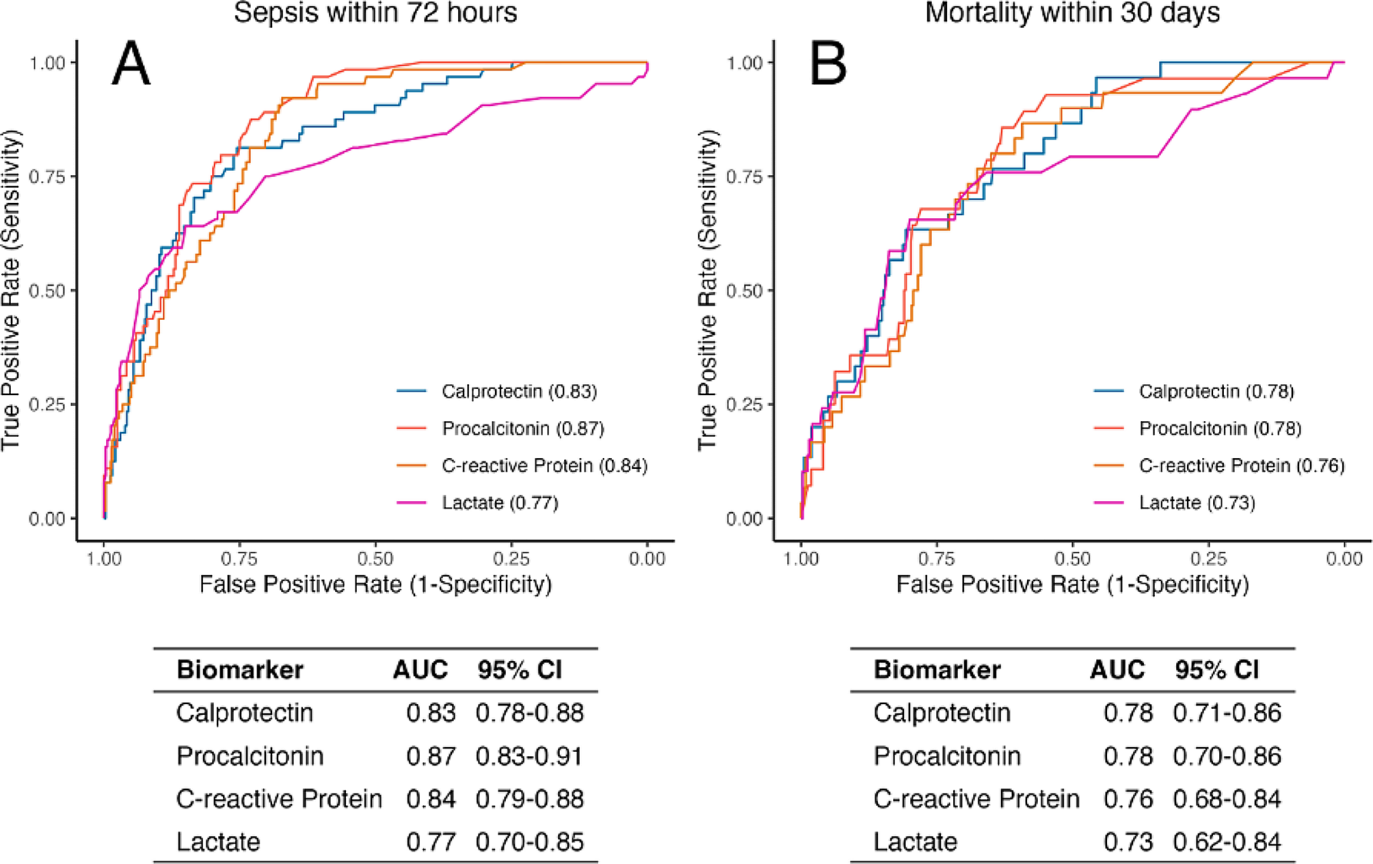
AUROCs for predicting sepsis and 30-day mortality. Performance of calprotectin in predicting (**A**) the occurrence of sepsis within 72 h of presentation, and (**B**) 30-day mortality, as compared to procalcitonin, CRP, and lactate. AUC = Area Under the Curve; CI = Confidence Interval

Patients adjudicated to have both an acute infection, as well as multi-organ dysfunction within 72 h of presentation, were considered to have sepsis, as outlined in Fig. 3. In total, 64 (16.2%) of patients were considered to have sepsis. Calprotectin was able to identify these patients with an AUROC of 0.83 (95%-CI = 0.78–0.88), similar to CRP, procalcitonin, and lactate (all *p*-value ≥ 0.05), as shown in Fig. 4. For 30-day mortality prediction, calprotectin had an AUROC of 0.78 (0.71–0.86), similar to 0.78 (0.70–0.86) for procalcitonin, 0.76 (0.68–0.84) for CRP, and 0.73 (0.62–0.84) for lactate.

### Visual analysis of calprotectin in sepsis

**Fig. 5:**
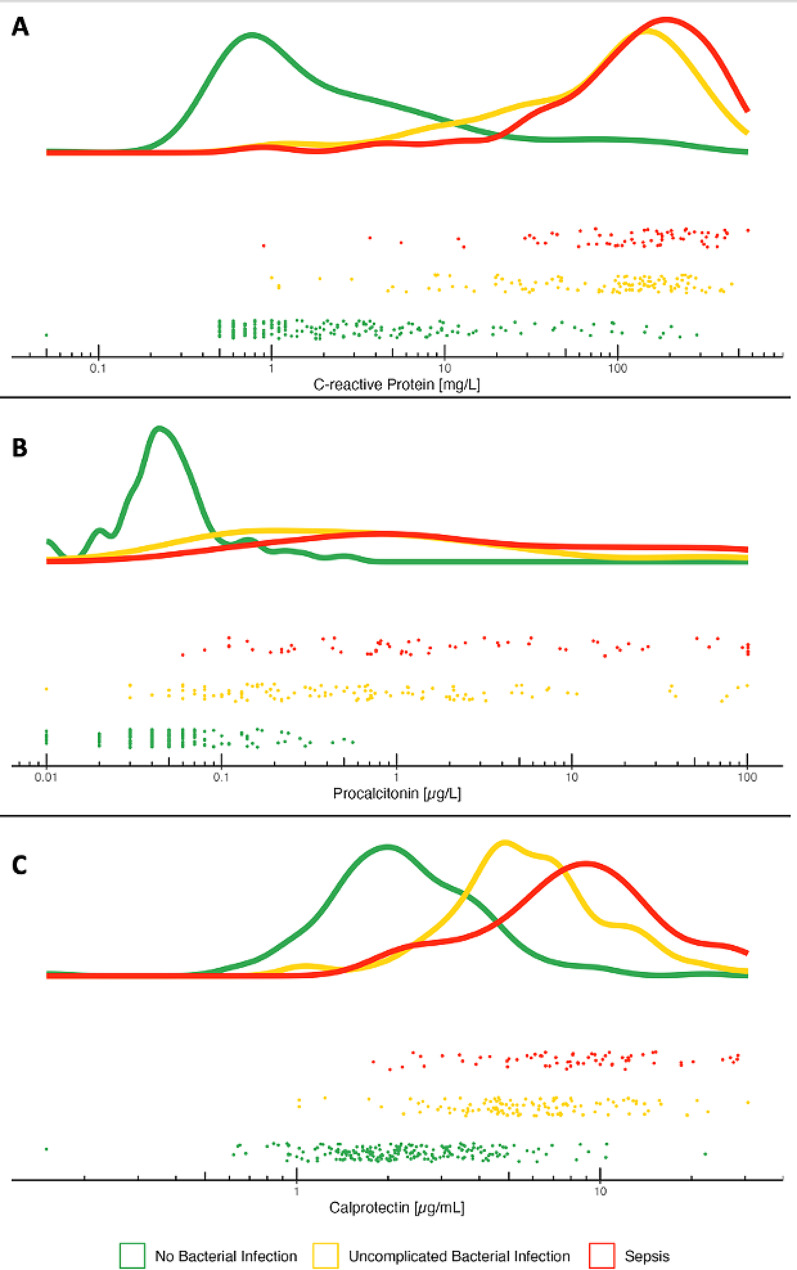
Biomarkers for non-bacterial, uncomplicated bacterial, and septic patients. This figure shows a visual analysis of the distribution of biomarkers related to outcome. Density and jitter plots of (**A**) C-reactive protein, (**B**) procalcitonin, and (**C**) calprotectin are shown on a logarithmic scale for patients without bacterial infections (green), with uncomplicated bacterial infections (yellow), and with sepsis (red). Each point in the jitter plots reflects the biomarker concentration of a single patient, segmented by the outcomes of the patient. The density plots are normalized to all have equal areas.

In a visual analysis shown in Fig. 5, the cohort was divided into three clinically relevant groups: (1) patients with no infection; (2) patients with bacterial infection but without multi-organ dysfunction; (3) patients with sepsis (infection and multi-organ dysfunction). For each group, the distribution of calprotectin, procalcitonin, and CRP was plotted on a logarithmic scale. On average, higher calprotectin concentrations were observed in septic patients than in those with non-septic bacterial infections, with the latter showing higher concentrations than patients without any infection.

## Discussion

In this study we investigated calprotectin as a biomarker for acute infections. We evaluated its accuracy for identifying bacterial infections overall, and in several common infection sites, its performance in clinically relevant patient subgroups defined by age and comorbidities, and in identifying and distinguishing clinically relevant outcomes, such as sepsis and 30-day mortality. In order to reflect real-life use, our study design included as few exclusion criteria as possible. Our cohort was enrolled at the point of initial presentation to the emergency department in order to evaluate calprotectin’s diagnostic potential at the earliest possible time point in treatment decision-making.

### Calprotectin identifies bacterial infections

In patients with bacterial infections calprotectin was significantly elevated, compared to patients without bacterial infections, in line with previous research [28]. As a diagnostic test, calprotectin showed a notable accuracy with an AUROC of 0.90, indicating its value in distinguishing cases of bacterial infection from viral infections and non-infectious mimics. The performance of calprotectin was moderately lower than the established biomarkers CRP and procalcitonin. However, the high performance of these two markers, with AUROCs of 0.93 and 0.94, respectively, should be considered biased, as CRP and procalcitonin were among the leading parameters used by the adjudicators to determine the infection status of the patients. In a comparable cohort in which adjudicators were blinded to procalcitonin, the AUROC for procalcitonin was found to be between 0.69 and 0.70 [29, 30]. Considering that calprotectin only weakly correlated with procalcitonin, and the adjudicators were blinded to calprotectin, its accuracy can be considered independent from the procalcitonin concentration, and therefore from the adjudicator-bias. An AUROC of 0.90, therefore, can be considered notable.

### Calprotectin in viral infections

Among patients with viral infections, an increase compared to non-infections was also noted, albeit significantly less than that for bacterial infections and co-infections. In our cohort, of the 50 patients with viral- or co-infections, 21 were SARS-CoV-2 positive, of which only two had severe COVID-19 with multi-organ dysfunction. Increases of calprotectin in cases of severe SARS-CoV-2 infection, have been previously reported [16, 31, 32], and were also observed in these two patients (6.42 μg/mL and 14.91 μg/mL), although it should be noted that both cases also had a bacterial co-infection.

### Performance is robust across foci, gram-types, and subgroups

Calprotectin was significantly elevated in all assessed infections sites - thus, diagnostic value of calprotectin measurement seems not limited in clinical application to specific organ systems. Moreover, regarding age, immunosuppression, and comorbidities, no limitation of diagnostic performance was found. Noteworthy, among patients with diabetes, we noted an increase in performance for calprotectin, while those of CRP und procalcitonin both decreased. While the performance of infection markers in diabetics has previously been analysed [33], no comparison to non-diabetics exists and the observed trends warrant further investigation. Among patients with bloodstream infections, gram-positive and gram-negative bacteria both induced an equally high rise in concentration. For procalcitonin, on the other hand, a significant difference in increase was noted, with gram-positive bacteria eliciting a weaker response than gram-negative bacteria, a finding in line with previous research [34]. In calprotectin the similar results in both types indicate a consistent performance independent of the causative bacteria. Together these findings show that an elevated calprotectin together with a clinical suspicion of an infection is a strong and robust indicator of bacterial infection and might assist physicians in deciding whether to give antibiotics.

### Calprotectin as a marker for prognosis in infection

Our cohort included 65 cases (16.5%) of multi-organ dysfunction within 72 h, and 30 patients (7.6%) died within 30 days of enrolment. For predicting sepsis, defined as multi-organ dysfunction and infection, and 30-day mortality calprotectin showed a similar performance to procalcitonin and CRP. This indicates the ability of calprotectin to not only identify bacterial infections, but also to correlate with the severity and outcome of the infection, as highlighted in Fig. 5. It is important, however, to highlight, that our study was neither designed nor powered to prove this effect. In this figure, the visual separation of the different peaks between the three groups (no infection, uncomplicated bacterial infection, and sepsis), especially between sepsis and infection. This might indicate an interesting and clinically valuable characteristic of calprotectin. Figures 4 and 5 show that calprotectin, to a certain extent, not only increases in cases of bacterial infection but also correlates with the infection severity.

### Value of blood calprotectin in clinical practice

In summary, calprotectin shows clear promise as a biomarker for bacterial infections and sepsis. Circulating calprotectin is already available on high throughput platforms in several routine clinical laboratories, used for diagnosing and monitoring inflammatory conditions such as rheumatoid arthritis and multiple sclerosis. This availability offers a significant benefit to many experimental markers for which no established assays exist. Therefore, calprotectin may well add value to ED clinical practice by being used in conjunction with the established markers procalcitonin and CRP.

Due to the heterogeneity of sepsis a universal routine single biomarker may not ever exist [35]. The future of characterizing and risk stratifying bacterial infections, and thus the future of sepsis diagnostics, may therefore lie in measuring multiple biomarkers and combining the results. This approach could involve using machine learning to calculate the probability of sepsis or the combined evaluation of markers by treating physicians [35, 36]. In fact, the observed orthogonality of calprotectin to procalcitonin and CRP in this cohort may indicate that a combined classifier could outperform the markers individually.

### Strength and limitations

Our study has strengths and limitations. The key strength is the large, prospective cohort from a real-life ED, enrolled with as few exclusion criteria as possible in order to generate a diverse and heterogenous sample. This is seen by the high rates of relevant comorbidities such as cancer and immunosuppression, as well as elderly patients and patients with adverse outcomes – all critically important patient groups which are often overlooked in sepsis research [37]. Key limitations of the trial are the monocentric design and the limited possibility of direct comparison to standard markers, procalcitonin and CRP, which were required for the adjudication process to maximize the accuracy. The observation, that calprotectin nevertheless produces similar accuracies to CRP and procalcitonin therefore shows its potential. Furthermore, the statistical power of the exploratory subgroup analysis was low and requires further research. Finally, longitudinal measurements would allow for more in-depth analysis into the kinetics of this important immune signaling protein but were not evaluated in this trial.

## Conclusion

In conclusion, this trial showed notable accuracy of blood calprotectin for all of the clinically relevant endpoints and across key subpopulations. Given that blood calprotectin is routinely measured for other indications, it is therefore already available in many clinical laboratories. Combined with its robust performance, this marker could complement the current diagnostic repertoire for patients with acute bacterial infections and sepsis for improved diagnosis and treatment decisions.

### Electronic supplementary material

Below is the link to the electronic supplementary material.

Supplementary Material 1

## Data Availability

The datasets used and/or analysed during the current study available from the corresponding author on reasonable request.

## Abbreviations

AUC: Area Under the Curve
AUROC: Area Under the Receiver Operating Characteristic
BP: Blood Pressure
BSI: Blood Stream Infection
CASCADE: Calprotectin in Acute Infections and Sepsis for Prognosis, Characterisation, and Diagnosis in the Emergency Department
CI: Confidence Interval
COPD: Chronic Obstructive Pulmonary Disease
CRP: C-reactive Protein
DRKS: German Register for Clinical Trials
ED: Emergency Department
ICU: Intensive Care Unit
IQR: Interquartile Range
MOD: Multi-Organ Dysfunction
ROC: Receiver Operating Characteristic
SOFA: Sequential Organ Failure Assessment
WBC: White Blood Count
