# Supplementary material for: Blood calprotectin as a biomarker for infection and sepsis – the prospective CASCADE trial
Source: BMC Infect Dis. 2024 May 16;24:496. doi: 10.1186/s12879-024-09394-x (PMC11100246; doi:10.1186/s12879-024-09394-x)

# Supplemental Content: Blood Calprotectin as a Biomarker for Infection and Sepsis – the Prospective CASCADE Trial

Eva Diehl-Wiesenecker^1^*, Noa Galtung^1^*, Johannes Dickescheid^1^, Monika Prpic^2^, Rajan Somasundaram^1^, Kai Kappert^2, 3^, Wolfgang Bauer^1^‡

# Table S1

|  | Infection Suspected  (N =194) | No Infection Suspected  (N = 201) | All  (N = 395) |
| --- | --- | --- | --- |
| **Demographics** |  |  |  |
| Age [years] | 75.5 (60.0, 82.8) | 74.0 (64.0, 81.0) | 75.0 (63.0, 82.0) |
| Female | 81 (41.8%) | 92 (45.8%) | 173 (43.8%) |
| Vital Signs |  |  |  |
| Respiratory Rate [/min] | 20 (16, 25) | 16 (14, 17) | 16 (14, 21) |
| Systolic Blood Pressure [mmHg] | 126 (106, 142) | 145 (130, 160) | 136 (119, 153) |
| SpO2 [%] | 96 (93, 98) | 98 (97, 100) | 98 (95, 99) |
| Temperature [°C] | 37.8 (36.7, 38.8) | 36.4 (36.1, 36.7) | 36.7 (36.3, 37.8) |
| Heart Rate [/min] | 99 (84, 113) | 78 (68, 91) | 87 (74, 104) |
| **Comorbidity** |  |  |  |
| Malignancy | 43 (22.2%) | 47 (23.4%) | 90 (22.8%) |
| Type 2 Diabetes | 47 (24.2%) | 28 (13.9%) | 75 (19.0%) |
| COPD | 22 (11.3%) | 18 (9.0%) | 40 (10.1%) |
| Immunosuppression | 22 (11.4%) | 8 (4.0%) | 30 (7.6%) |
| **Biomarkers** |  |  |  |
| Calprotectin [µg/mL] | 6.4 (4.3, 9.7) | 2.1 (1.5, 3.0) | 3.5 (2.0, 6.4) |
| Procalcitonin [µg/L] | 0.6 (0.1, 2.2) | 0.1 (0.0, 0.1) | 0.1 (0.1, 0.7) |
| C-reactive protein [mg/L] | 114.2 (41.9, 203.5) | 1.1 (0.7, 3.5) | 10.3 (1.1, 118.5) |
| White Blood Count [/nL] | 12.9 (9.1, 18.4) | 7.6 (6.2, 8.9) | 8.9 (6.8, 13.6) |
| Lactate [mg/dL] | 18.0 (13.0, 27.9) | 13.0 (11.0, 17.0) | 15.0 (11.0, 22.0) |
| **Outcomes** |  |  |  |
| Bacterial Infection | 145 (74.7%) | 11 (5.5%) | 156 (39.5%) |
| Viral Infection | 21 (10.8%) | 9 (4.5%) | 30 (7.6%) |
| Co-Infection | 20 (10.3%) | 0 (0.0%) | 20 (5.1%) |
| No Infection | 8 (4.1%) | 181 (90.0%) | 189 (47.8%) |
| MOF within 72h | 61 (31.4%) | 4 (2.0%) | 65 (16.5%) |
| Sepsis within 72h | 61 (31.4%) | 3 (1.5%) | 64 (16.2%) |
| ICU within 72h | 48 (24.7%) | 15 (7.5%) | 63 (15.9%) |
| Death by Follow-up 30d | 24 (12.5%) | 6 (3.0%) | 30 (7.6%) |

Table S1. Baseline Characteristics at enrolment by cohort. Abbreviations: SpO2 = Oxygen saturation; GCS = Glasgow Coma Scale; COPD = Chronic Obstructive Pulmonary Disease; MOF = Multi-Organ Failure; ICU = Intensive Care Unit.

# Figure S1. Correlation Plots of Calprotectin vs. Procalcitonin and CRP


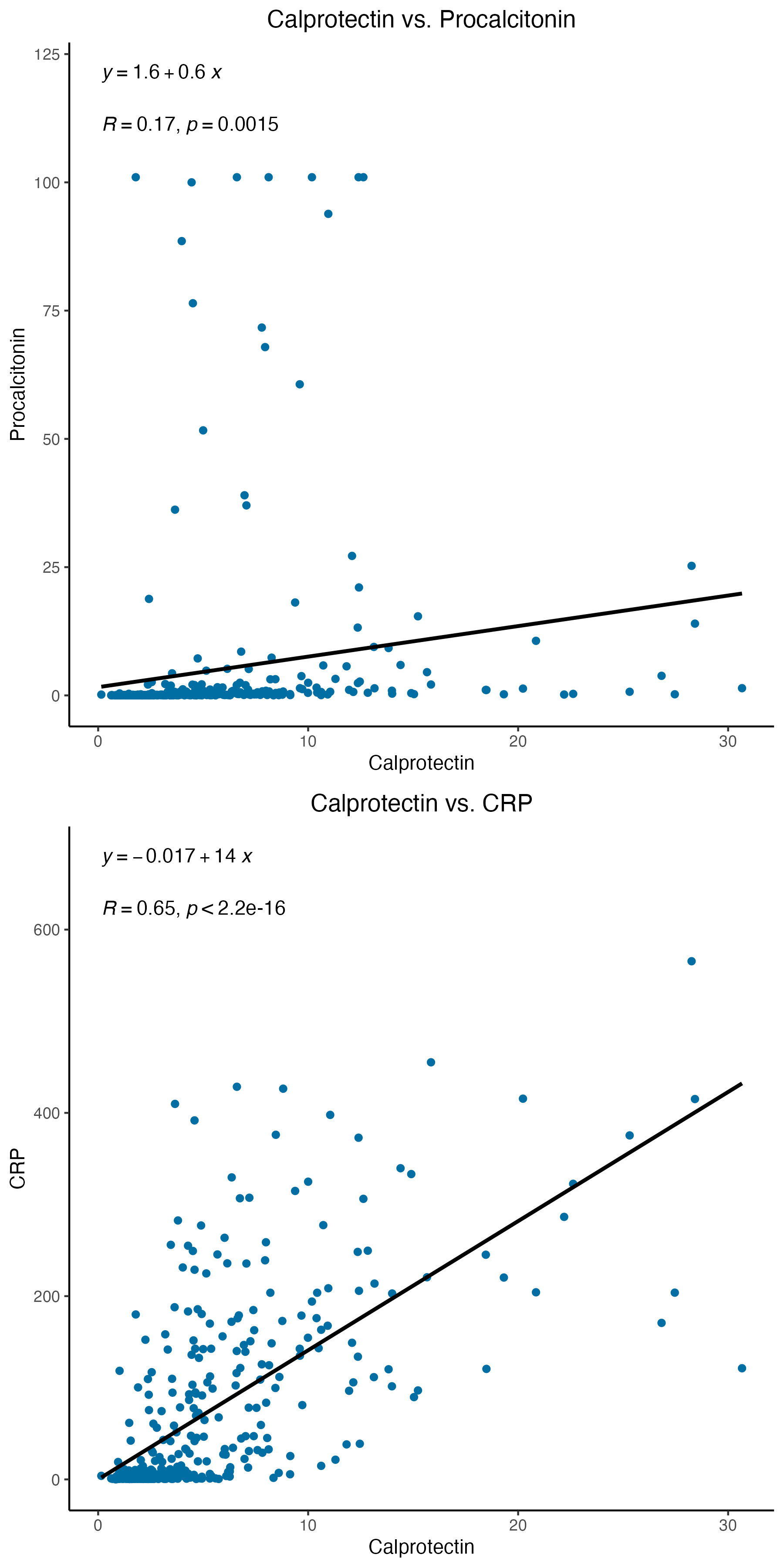


# Figure S2. Distribution of calprotectin concentrations by adjudicated infection status


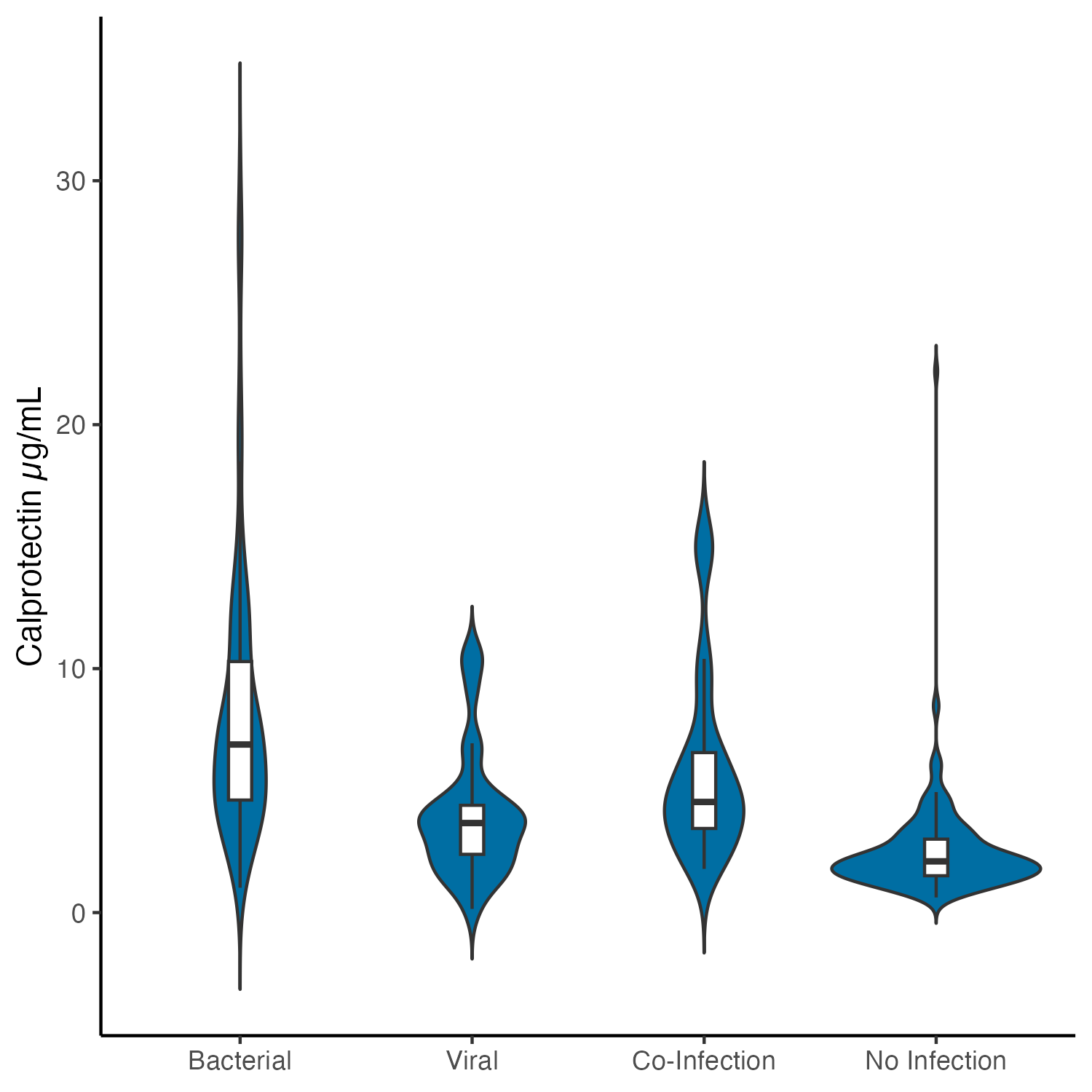


Distribution of calprotectin concentrations by adjudicated infection status.

# Figure S3. Receiver Operating Characteristics for Bacterial Infection


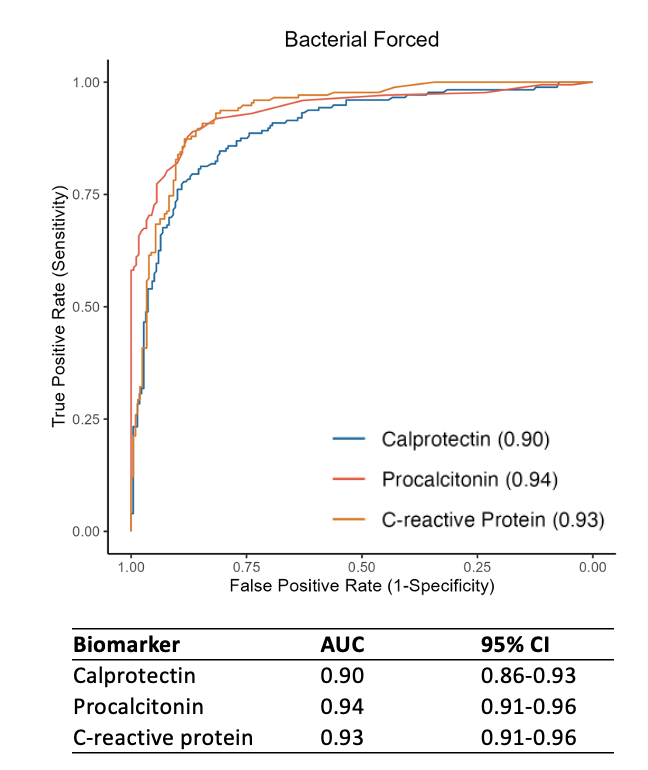


Receiver Operating Characteristic of calprotectin, procalcitonin and C-reactive protein in identifying bacterial infections. This includes bacterial-viral co-infections as bacterial, and both non-infectious and viral conditions as non-bacterial. Since C-reactive protein and procalcitonin were used in the adjudication of the endpoint, these are not considered valid comparators. The Areas Under the Curves, for all three parameters, are shown, including 95% confidence intervals (CI).

# Figure S4. Receiver Operating Characteristic Curves for Calprotectin for Diagnosing Bacterial Infections in Key Subpopulations


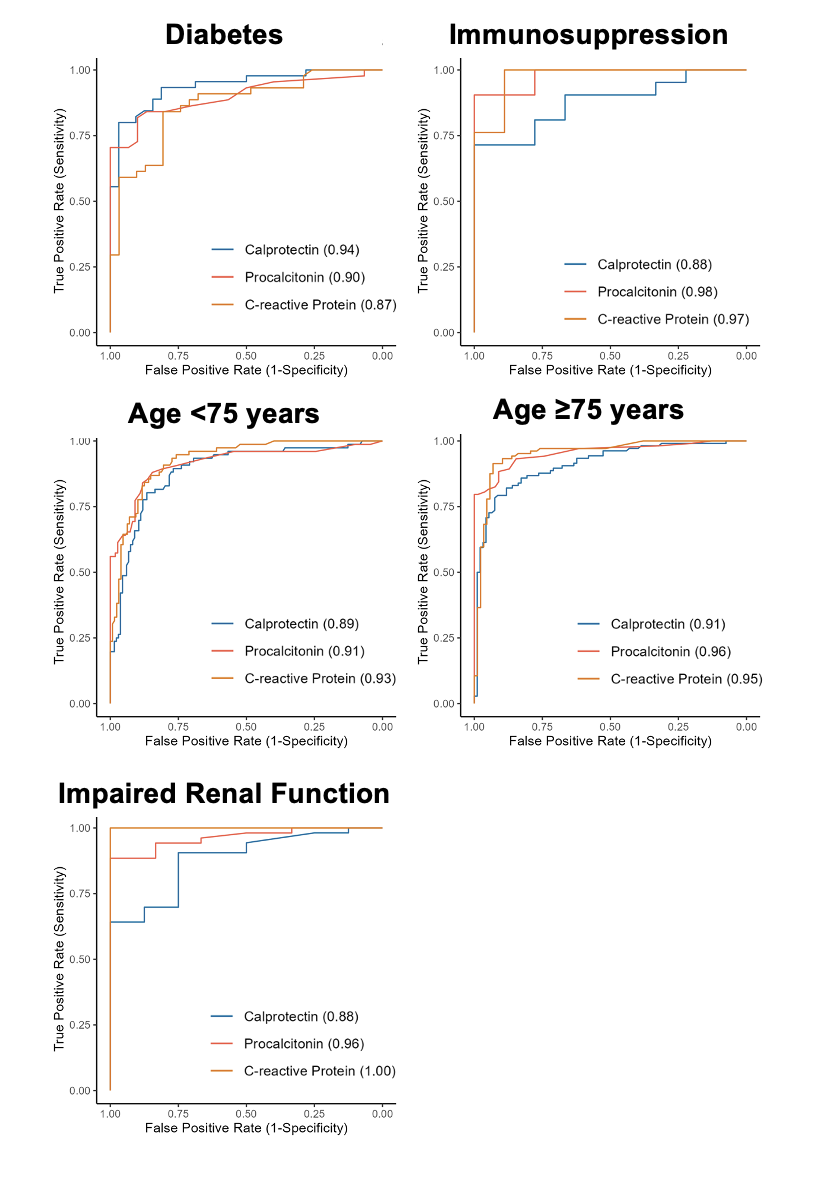


Performance of calprotectin, procalcitonin and c-reactive protein for diagnosing bacterial infections in key subpopulations.

# Supplemental Methods

In a subset of 29 samples, calprotectin measurements were performed in serum samples from serum gel vacuum tubes due to a lack of available heparin plasma samples. To this end, we measured calprotectin concentrations in both heparin plasma and serum samples taken simultaneously from a sub-cohort of 30 patients. These were then used to generate a linear model (R^2^ = 0.72, p < 0.001), as shown in the figure below. The resulting function could then be used as corrective equation to convert the serum concentrations of the 29 patients without plasma samples into plasma-equivalents. Further, the statistical analyses shown in the publication were also performed under the exclusion of these values, with no significant changes to the resulting accuracies.


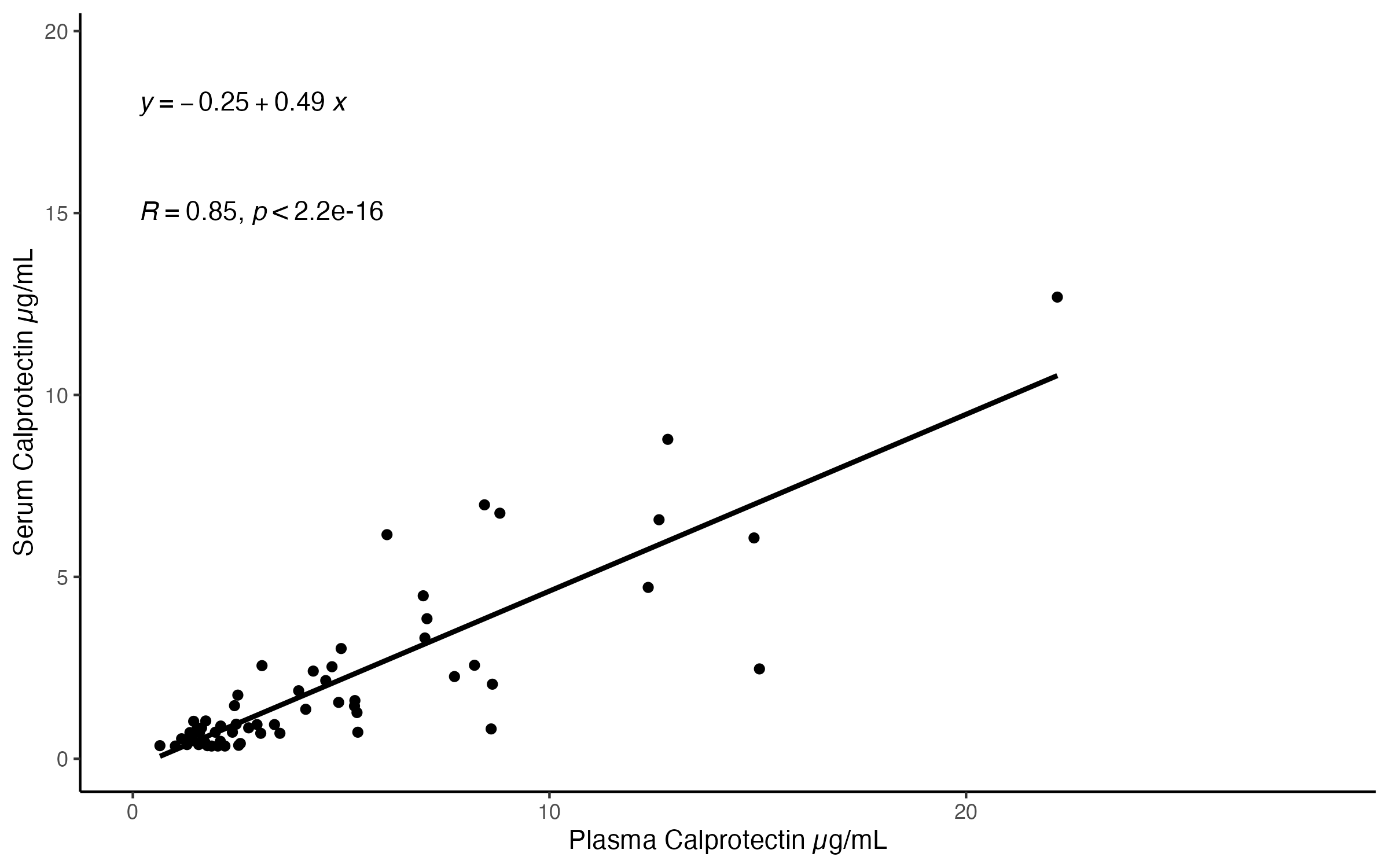

Supplement: Supplementary file 1 — Supplementary Material 1 [file 12879_2024_9394_MOESM1_ESM.docx]
